# Clutter-Masked Waveform Design for LPI/LPD Radarcom Signal Encoding

**DOI:** 10.3390/s21020631

**Published:** 2021-01-18

**Authors:** Richard Washington, Brenton Bischof, Dmitriy Garmatyuk, Saba Mudaliar

**Affiliations:** 1 Department of Electrical and Computer Engineering, Miami University, Oxford, OH 45056, USA; washinr3@miamioh.edu (R.W.); bischobc@miamioh.edu (B.B.); 2 U.S. Air Force Research Laboratory, Wright-Patterson AFB, Fairborn, OH 45433, USA; saba.mudaliar@us.af.mil

**Keywords:** clutter modeling, radar-communication fusion, software-defined radar, UWB radar

## Abstract

In this work we propose a method of in situ clutter deconvolution and modeling using experimentally obtained UWB radar data. The obtained clutter models are then used for random sequence encoding of radar-communication (radarcom) signals to achieve clutter-masked transmissions and improve communication security. We present the results of clutter modeling from the laboratory data obtained with the software-defined radar system. We then show that such clutter-masked radarcom signals generated using the local clutter model are highly likely to be interpreted as just clutter returns by an unauthorized interceptor. We also present the results of communication and radar performance of these radarcom signals and contrast them with those obtained using a linear frequency modulated waveform. It is shown that the proposed radarcom design method has high potential to achieve secure communications in adversarial conditions, while simultaneously addressing radar sensing needs.

## 1. Introduction

Combination of radar sensing and wireless communications—sometimes dubbed “radarcom”—is a relatively new topic in radio frequency (RF) signal and system design. The main motivation for such a fusion is usually threefold: (a) The desire to reduce size, weight, power, and cost (SWaP-C) requirements; (b) the need to resolve spectrum use constraints arising from an ever-growing list of desired system functionalities; and (c) the desire to increase covertness of operations. The first two items are addressed via “intelligent” waveform design, which allows for achieving acceptable performance of both functionalities [1,2]. The third item is usually proposed to be attained by embedding a communication signal into the high-power radar transmissions [3], or such pulsed radar transmissions can also be used to energize RF circuits in communication tags preplaced in the area [4]. Also, in [4], RF tag transmissions are designed to appear similar to the radar backscatter, or clutter.

In our previous work, we investigated the performance of the random sequence encoding (RSE) method of waveform design, where the transmissions could be derived from a clutter model [5]. This was achieved via a novel algorithm in which the communication data are encoded as a parameter of a particular random distribution, whereas the radarcom signal is then created using samples of the thus distributed random process. We used Weibull distribution for those purposes—however, the low probability of intercept/detect (LPI/LPD) potential of the transmissions can be further enhanced if an actual in situ clutter distribution model is implemented.

Clutter characterization and modeling are, thus, important topics for covert radarcom signal design. Commonly, in radar surveillance, clutter is regarded as a detrimental effect that lowers the capabilities of the sensor, leading to performance degradation. For this reason, clutter suppression/removal techniques have been studied, e.g., space-time adaptive processing (STAP) [6]. To better understand the impact of clutter and thus be more efficient in its removal, clutter modeling has been investigated, e.g., in [7] the authors used deconvolution to remove sea clutter from the radar returns based on the theoretical model of the former. In [8], cumulative disturbance was modeled as combination of clutter and noise. In [9,10], methods to model clutter as spherically invariant random vectors (SIRV) were introduced. In none of those works, however, were any attempts to separate noise from the clutter model made. This may not be a requirement for subsequent clutter removal algorithms, still other uses for clutter modeling may necessitate such a delineation.

There have been a variety of waveforms suitable for sensing, and communications studied recently as radarcom schemes have gained attention. In [11], noise-like radarcom signals were introduced using the Lorenz chaos system, with a flattened spectrum. This resulted in peak sidelobes (PSL) of −32 dB over a bandwidth of 500 MHz. The signal included 300 communication symbols and a symbol length of 100. The Lorenz chaos approach was also used in [12] to create controlled chaos-based frequency modulated (CCBFM) waveforms PSL at −17 dB. The bit error rate (BER) was measured to be 0.125 at an energy-per-bit to noise spectral density ratio (E_b_/N) between −5 dB and 2 dB, and the BER was 0.001 at a (E_b_/N) of 10 dB. While the chaos-based waveforms are claimed to have good LPI/LPD properties, there were no quantifiable results presented in that regard yet. Also, since such signals are not part of the natural RF environment and may go above the noise floor, it may be easier for an interceptor to identify this part of the spectrum as potentially containing information. In contrast, our proposed method aims to create transmissions that represent the in situ clutter, leading to much higher likelihood that an interceptor will classify such intercepts as clutter returns and not communication data. Further differences between these approaches and our proposed method are that the Lorenz scheme in [11] uses well known modulation such as differential chaos shift keying (DCSK), which makes it more likely to be deciphered; additionally, the bits per seconds (bps) figure in [12] was significantly lower than what orthogonal frequency division multiplexing (OFDM) can provide. In [13], phase modulated continuous wave (PMCW) and OFDM access (OFDMA) were tested. OFDMA differs from OFDM in allocating users in both time and frequency instead of just time. Both methods had PSL of −9 dB and sidelobe floors of −10 dB. In [14], a survey was conducted of various joint radar and communication schemes; one notable version combined the linear frequency modulated (LFM) chirp and OFDM. The only LPI/LPD-capable radarcom approaches noted in that work were RF tag-based methods, e.g., [4].

The idea of using machine learning (ML) for clutter identification has also been investigated in recent years. In [15], the Ozturk algorithm was tested alongside the sparse recovery method of batch orthogonal matching pursuit (BOMP) algorithm. These methods were tested on Log-Normal and Weibull distributions. BOMP outperformed the Ozturk method with an accuracy of between 74–95% compared to the Ozturk’s 22–42% accuracy. Samples ranged from 300 to 2800, while dictionary sizes ranged from 500–2500. A non-supervised ML method was used in [16] to distinguish between two sets of 100 random vectors (each with 100 elements) from two different Spherically Invariant Random Vectors (SIRV). It was able to do so with 86% accuracy. In our work, we will show that verifying a heuristic estimate of the distribution from experimental data is a computationally efficient way to determine the clutter mode when compared to ML techniques.

The purpose of this work is to introduce a method of clutter model construction, based on real experimental measurements of radar returns from which noise contribution is removed—then, apply the results to the construction of clutter-masked transmissions, which are used for radar sensing and data communication purposes simultaneously. These transmissions are deemed secure in the sense that an unauthorized receiver will likely interpret these intercepts as local clutter returns and will not attempt decoding them for their information content.

The rest of the paper is organized as follows: we briefly review the RSE method of radarcom signal design in Section 2; then, we introduce our approach to clutter and noise deconvolution from real radar data in Section 3; we present our results of clutter modeling in Section 4; Section 5 discusses results of performance evaluation of the created clutter-masked radarcom signals with regards to: (a) interceptor classification of these signals as clutter returns; (b) communication efficiency; and (c) synthetic aperture radar (SAR) image reconstruction with backprojection. Finally, we offer concluding remarks and suggestions for future work in Section 6.

## 2. RSE Approach to Signal Design

We use the OFDM modulation scheme to create either pulsed or continuous wave (CW) radarcom sampled signal, with samples $s_{n}$:

(1) $$s_{n}=\frac{1}{2L+1}\sum_{m=1}^{2L+1}S(m)\cdot e^{j2\pi \frac{\left(m-1)(n-1\right)}{2L+1}},$$

where *L* is the total number of OFDM sub-carriers and S(m) is an amplitude coefficient of the *m*^th^ OFDM sub-carrier. These amplitude coefficients are proposed to be drawn from a random process with a probability density function (PDF) known to all authorized platforms.

In [5], we selected Weibull-distributed random process with probability density function (PDF)f(x;λ,k), where *λ* is the scale parameter and *k* is the shape parameter. Assuming that the shape parameter *k* is selected arbitrarily and is known to the authorized platforms only, we proposed to encode communication data using the scale parameter *λ*. For simplicity, we formulate that correctly recovering an estimate of *λ* by an authorized receiver achieves our goal of the communication data reception. An authorized receiver can perform this estimation using a simple expression [5,17]:

(2) $$\hat{\lambda }=\frac{E\left[\hat{S}\right]}{\Gamma \left(1+\frac{1}{k}\right)},$$

where $E\left[\hat{S}\right]$ is mathematical expectation of the vector $\hat{S}$ containing estimates of the received signal’s OFDM sub-carrier amplitudes, and Γ is the gamma function. In contrast, an unauthorized interceptor without the knowledge of *k* will need to perform estimation of that parameter first, which requires solving a non-linear equation that is highly susceptible to disturbances, such as additive noise:

(3) $${\hat{k}}^{-1}=\frac{\sum_{m=1}^{2L+1}{\left(\hat{S}(m)\right)}^{k}\ln\left(\hat{S}(m)\right)}{\sum_{m=1}^{2L+1}{\left(\hat{S}(m)\right)}^{k}}-\frac{1}{2L+1}\sum_{m=1}^{2L+1}\ln\left(\hat{S}(m)\right),$$

where $\hat{S}(m)$ is the estimate of the *m*^th^ OFDM sub-carrier amplitude. We showed in [18] that an unauthorized interceptor would need signal-to-noise ratio (SNR) of intercepts to be above +20 dB to achieve acceptable accuracy in estimating *k*—thus reconstructing the data.

Therefore, the RSE scheme is based upon using one of the parameters of a PDF for encoding information, and another parameter (or parameters) as a “key” with which the data can be reconstructed. The same signal that is generated in this fashion can be used for radar sensing. This approach is different from radar-embedded communications, as well as from RF tag-based one-way communications enabled by using radar beam energy.

The security of this scheme can be further enhanced if in place of an arbitrary distribution an actual local clutter model was used, which is the focus of the next sections.

## 3. Clutter Distribution Deconvolution from Noise

Let us consider a scenario in which an ultra-wideband (UWB) radar illuminates a segment of a terrain with an underlying clutter model. The received signal can then be represented as:

(4) $$S_{R}=C+N,$$

where *C* is contribution from clutter and *N* is noise contribution, and distributions of each are not known, although it is known that clutter and noise are statistically independent. We then identify two cases:

Case 1: Radar receiver is on, but the transmitter is off.

Case 2: Both the transmitter and receiver are on.

In Case 1, the only received component with a non-zero value in (4) is *N*. In Case 2, the return is the sum of independent random variables *C* and *N*. In the actual system implementation, the received signal is recorded in terms of its magnitude samples [5]. Owing to the independence of *C* and *N*, we then formulate the expression for their joint PDF, expressed via their individual pdf’s:

(5) $$p_{C+N}\left(x\right)=p_{C}(x)⊗p_{N}(x),$$

where the ⊗ sign denotes convolution.

Let us introduce characteristic function of the joint clutter and noise disturbance, which, by definition [19], is the Fourier transform of the corresponding pdf:

(6) $$ℑ\left(p_{C+N}\left(x\right)\right)=\varphi_{C+N}\left(\omega \right)=\int_{-\infty }^{\infty }p_{C+N}\left(x\right)\cdot e^{j\omega x}dx.$$

Following radar return collection in Case 1, we can obtain the characteristic function of noise-only returns, $\varphi_{N}\left(\omega \right)$; similarly, Case 2 will yield $\varphi_{C+N}\left(\omega \right)$. Then, the problem of finding clutter-only pdf from the collected data reduces to solving for $p_{C}(x)$ via deconvolution:

(7) $$p_{C}\left(x\right)={ℑ}^{-1}\left(\frac{\varphi_{C+N}\left(\omega \right)}{\varphi_{N}\left(\omega \right)}\right),$$

where ${ℑ}^{-1}$ denotes inverse Fourier transform (IFT).

**Figure 1:**
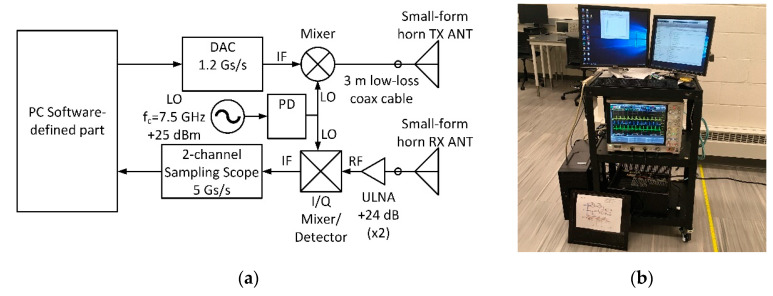
Lab setup: (**a**) Ultra-wideband (UWB) software-defined radar system (SDRS) block diagram; (**b**) SDRS transceiver in laboratory setting.

Radar returns for Cases 1 and 2 were collected at an indoor laboratory location using UWB software-defined radar system (SDRS), described in detail in [20]. The indoor setting was an empty 10-meter-long hallway with concrete floor, walls, and ceiling. The system has useful baseband bandwidth of 600 MHz, which is determined by the sampling rate of the digital-to-analogue converter (DAC). Transmissions are performed at the center frequency of 7.5 GHz, which was selected due to cost, availability, and efficiency considerations pertaining to the local oscillator (LO) and the antennas. A block diagram of the SDRS is shown in Figure 1a and an image of SDRS with collocated transmitter and receiver antennas is shown in Figure 1b. We used our SDRS in monostatic configuration, where the transmit and receive antennas were collocated.

To comply with the U. S. Federal Communications Commission (FCC) requirements for maximum permitted emissions in UWB frequency range, the output power was limited to that provided by the LO input to the upconverting mixer, as shown in Figure 1a. This restriction limits the useful range of our experiments to approximately 5 m.

## 4. Clutter Modeling from Experimental Data

RSE OFDM signals were generated in the frequency domain and converted to the time domain as per Equation (1). The transmitted signals contained *L* = 16 subcarriers, each with a uniform amplitude weight of 1. Ten different trials were performed, with $2\cdot 10^{6}$ collected samples each. These data samples of a random process were composed of cumulative effects of clutter and noise. To collect samples of noise only, the transmitter was turned off while the receiver was on and the same quantity of data was recorded.

Once the noise and return signals’ samples were collected and converted to frequency domain, two sets of PDFs were created for each trial/dataset: (a) noise and “noise + clutter” PDFs obtained directly from the collected data by using the function *ksdensity* in MATLAB^®^ [21], and (b) noise and “noise + clutter” PDFs recreated from clutter pdf $p_{C}\left(x\right)$ found from (8). Based on the obtained data, we modeled the clutter in the frequency domain using the Arcsine distribution [22].

Then, we performed mean square error (MSE), normalized mean square error (NMSE), and normalized root mean square error (NRMSE) tests on the two sets of PDFs. The results of these tests are shown in Table 1. Note that 0 is the best value for MSE and 1 is the best value for NMSE and NRMSE. It is evident that the PDFs for same processes obtained in these two ways are well matched, which confirms the validity of our method.

The expressions for the inverse cumulative distribution function (CDF), PDF, and CDF of the Arcsine distribution are shown in Equations (8)–(10), respectively.

(8) $$F^{-1}\left(u\right)=b\sin\left({\left(\frac{u\pi }{2}\right)}^{2}\right),$$

(9) $$f\left(x\right)=\frac{1}{\pi \sqrt{x\left(x-b\right)}},$$

(10) $$F\left(x\right)=\frac{2}{\pi }\sin^{-1}\left(\frac{x}{b}\right).$$

## 5. Clutter-Masked Radarcom Waveform Performance Evaluation

### 5.1. Transmit Waveform Design

Once the underlying clutter model has been ascertained, our goal becomes to create radarcom signals that would appear like clutter returns to an unauthorized receiver. We define the general format of OFDM sub-carrier coefficients S(m) as:

(11) $$S\left(m\right)=W(m)\cdot e^{j\phi (m)},$$

where W(m) is the *m*^th^ sub-carrier magnitude, and ϕ(m) is its phase. We then aim to assign samples drawn from the clutter random process with the modeled PDF to W(m), and samples drawn from a uniformly distributed random process to ϕ(m).

In order to accomplish the above, we first generate random samples from the uniform distribution within the interval [0, 1], and input them into the Arcsine distribution’s inverse CDF (8), which then outputs random samples drawn from the Arcsine distribution. We used the uniform distribution with interval [0, 2π] to generate random phase samples ϕ(m), which is characteristic for clutter returns [23].

### 5.2. Intercepted Signal Classification by Unauthorized Receivers

Our local clutter model in the frequency domain was modeled with the Arcsine distribution with the b-parameter equal to 100, which was also used to create our transmitted signals, as per (1) and (11). We assume that an unauthorized interceptor will attempt to analyze our transmissions after gaining access to them. We also assume that the interceptor is aware of the frequency band used by our radar, as well as of the method of signal design using OFDM. The interceptor is expected to classify the received signals in order to determine whether they may also contain other useful data.

We model this classification process as goodness-of-fit (GoF) tests, which compare the intercepts to distributions of the local clutter with a slightly varying b-parameter, as well as to uniform and normal distributions. Any significant deviations of intercepts from the local clutter distribution would be regarded as those containing useful data. The GoF was evaluated by using the MSE, NRMSE, and NMSE for 200 intercepted signals. The results of these GoF tests are shown in the Table 2 below, where MSE = 0 indicates the closet fit and ∞ the worst; for NMSE and NRMSE, 1 indicates the closest fit and −∞ is the worst. All three methods demonstrate the agreement that the Arcsine distribution with b = 100 is the best fit, which means that the intercepted radarcom signal would be classified as clutter by an unauthorized platform.

### 5.3. Clutter-Masked Communication Performance

After establishing the method’s clutter-masking potential, we need to investigate the efficiency of communications. The *b*-parameter of the Arcsine distribution was used to encode communication data. Depending on the symbol being sent, the *b*-parameter was changed before being used in (8). Upon reception, this parameter was estimated using the relationship between it and the average value of received samples of OFDM sub-carrier amplitudes. This method was tested in simulation, as described below.

First, we created an arbitrary 168-bit binary signal. We assumed that the clutter in the area was described by Equations (8)–(10) where *b* ≈ 110. The mean of this distribution converges to

(12) $$E[W]=\frac{b}{2},$$

where **W** is a vector containing samples of OFDM sub-carrier coefficients from (11) and *E*[..] denotes mathematical expectation, or average value. We then set our *b-*parameter for use in radarcom signal generation to a value of 100 for bit value of 0, and to 120 for bit value of 1. An OFDM signal with 32 sub-carriers was generated for each bit using (1) with S(m) formed as per (11). Upon reception, a friendly platform used FFT to reconstruct the sub-carrier coefficients S(m) using FFT and extracting the magnitude estimates $\hat{W}$ only, via the process explained in [5]. Once these estimates were obtained, the value of the *k*^th^ bit $A_{k}$ was estimated using the vector ${\hat{W}}_{k}$ and the estimate of its corresponding *b*-parameter as:

(13) $${\hat{A}}_{k}=\left\{ \begin{array}{l} 0\;if\;{\hat{b}}_{k}=2E\left[{\hat{W}}_{k}\right]<55\\ 1\;if\;{\hat{b}}_{k}=2E\left[{\hat{W}}_{k}\right]>55 \end{array}\right..$$

**Figure 2:**
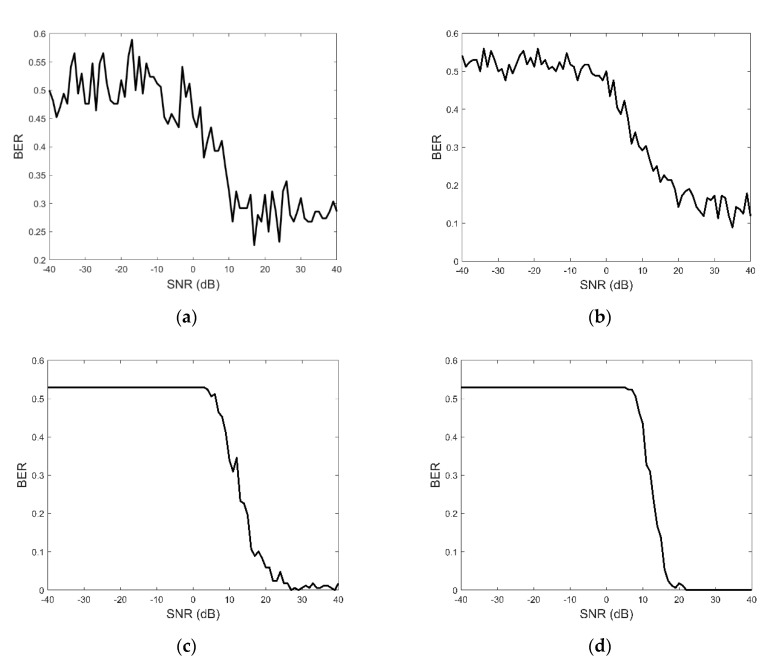
Bit error rate (BER) vs. signal-to-noise ratio (SNR) for clutter masked communications: (**a**) 1 Retransmission; (**b**) 10 Retransmissions; (**c**) 100 Retransmissions; (**d**) 300 Retransmissions.

Additive white Gaussian noise (AWGN) was added to the received clutter-masked signals and BER characteristics were computed for various signal-to-noise ratios (SNR). The resultant BER vs SNR plots are shown in Figure 2 for the cases of single transmission, as well as 10, 100, and 300 retransmissions per signal, where each retransmission carries the same bit sequence, but each waveform realization was different thanks to drawing the signal samples from the random distribution as described above. In absence of error control coding, or other improvements, we note that the obtained BER values are higher than those of modern broadband data communication schemes; as pointed out in [5], however, this is due to prioritizing transmission security over BER/throughput. Compared to [12,13], we note that the proposed scheme has poorer BER below +10 dB, but better bps potential and much stronger LPI/LPD characteristics.

### 5.4. Radar Performance

**Figure 3:**
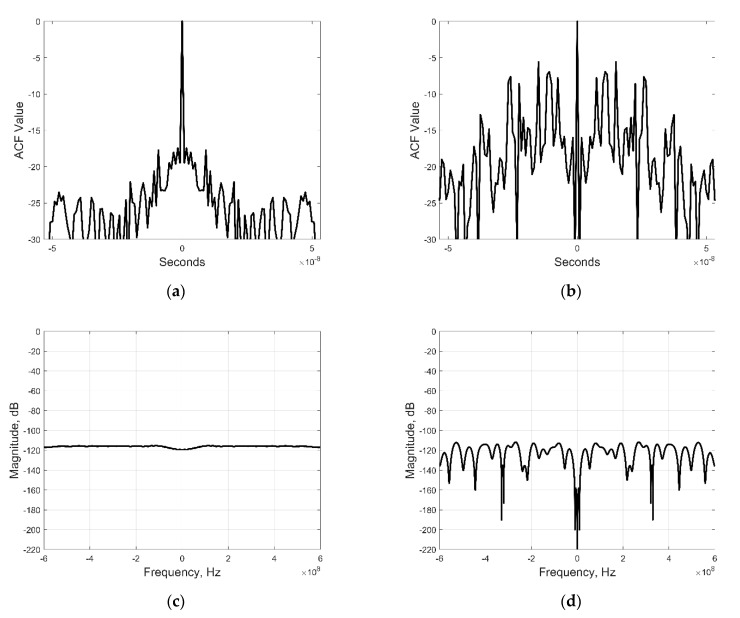
Radarcom and linear frequency modulated (LFM) signal illustrations: (**a**) Autocorrelation function (ACF) of LFM signal; (**b**) ACF of clutter-masked orthogonal frequency division multiplexing (OFDM) signal; (**c**) Frequency-domain representation of LFM signal; (**d**) Frequency-domain representation of clutter-masked OFDM signal.

To test the potential radar performance of the created radarcom signals, we computed their autocorrelation function (ACF) and contrasted it with ACF of a linear frequency modulated (LFM) signal with the same bandwidth and energy. These characteristics are shown in Figure 3 below. As expected, OFDM-generated signals exhibit worse ACF characteristics than those of LFM. This issue needs to be addressed for acceptable radarcom performance. One approach is to apply weights to the amplitude and phase factors in (12), as was investigated, e.g., in [24,25]. Also, while the main peak of clutter-masked OFDM is narrower than the OFDMA in [13], the sidelobes are higher than the examples in [11,12,13], which is the tradeoff made for enhancing the LPI/LPD capabilities.

**Figure 4:**
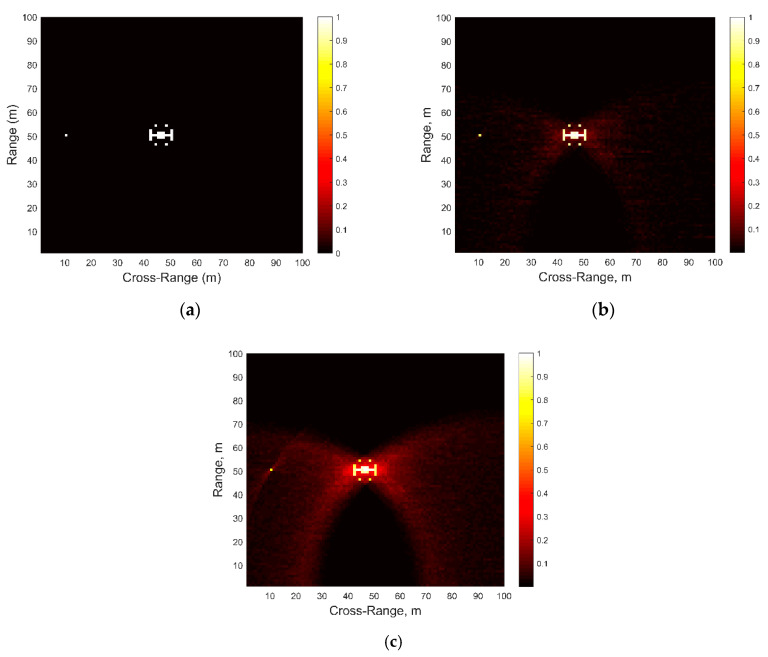
Backprojection via synthetic aperture radar (SAR): (**a**) Target Scene; (**b**) Return image of LFM signal; (**c**) Return image of clutter-masked OFDM signals.

We also tested the two signals in synthetic aperture radar (SAR) simulations, where the method of time-domain backprojection was used for image reconstruction [26]. This method was chosen due to its wide use and robustness, although it is possible that other SAR image reconstruction approaches could provide for better performance and/or computational efficiency. The original simulated scene, shown in Figure 4a, contained an 18-pixel “target” model at (47, 50) coordinates in cross-range and range, and a single-point “target” at (10, 50). The “target” pixels were assigned the value of 1, while the background pixels had value of 0. The backprojection algorithm was implemented under “stop-and-go” approximation [26]. The grid contained same number of points in range and cross-range for all simulations and the SNR was fixed at a very high value of approximately +75 dB so as to eliminate the effects of noise and focus on image reconstruction abilities of the two types of signals only: (a) LFM, and (b) Clutter-masked OFDM.

Figure 4b,c show the reconstructed SAR images with LFM and clutter-masked OFDM signals, respectively. We observe that while the higher sidelobes of OFDM signals contribute to the unwanted image obscuration, both target positions can still be ascertained very well.

## 6. Conclusions

In this paper we have introduced a method for clutter decorrelation from UWB radar returns, provided motivation for using this approach to improve LPI/LPD characteristics of RSE for radarcom signal design, and tested the created clutter-masked RSE radarcom waveforms in simulations for their performance in communications and radar. Our clutter decorrelation and clutter model design approach were tested experimentally with a SDRS in indoor laboratory conditions. We simulated the performance of such signals in noise and found that a friendly receiver achieves BER below 10% for SNR values above approximately 15 dB with 300 retransmissions, each of which employed randomized signals, which would be interpreted as clutter returns by an unauthorized interceptor using MSE, NMSE, and/or NRMSE for intercept classification. Additionally, we showed the potential for these signals to be used in radar applications, such as SAR.

Our future efforts will focus on rigorous modeling of the unauthorized receiver’s classification of intercepted radarcom signals, improving ACF characteristics of the created signals, modeling clutter through ML techniques, and analyzing other potential clutter distributions for the purpose of applying them to radarcom signal design.

## Figures and Tables

**Table 1:** Comparison of clutter and Arcsine distribution.

Probability Density Function(PDF) Type	Mean Square Error (MSE)	Normalized Mean Square Error (NMSE)	Normalized Root Mean Square Error (NRMSE)
Directly Obtained (Noise+Clutter)	6.758 × $10^{-6}$	0.9985	0.9616
Recreated (Noise+Clutter)	4.677 × $10^{-6}$	0.9990	0.9677

**Table 2:** Table of Probability Distribution Comparisons.

PDF Type	MSE	NMSE	NRMSE
Arcsine (b = 100)	3.125 × $10^{-6}$	0.9974	0.9493
Arcsine (b = 101)	3.933 × $10^{-6}$	0.9967	0.9429
Arcsine (b = 99)	5.447 × $10^{-6}$	0.9956	0.9336
Normal (μ = 50; σ = 12.5)	0.0132	−0.1068	−0.0521
Uniform [0.1, 100]	4.125 × $10^{-4}$	0.7106	0.4620

## Data Availability

The data presented in this study are available on request from the corresponding author.
